# Synthesis of Petal-Like MnO_2_ Nanosheets on Hollow Fe_3_O_4_ Nanospheres for Heterogeneous Photocatalysis of Biotreated Papermaking Effluent

**DOI:** 10.3390/ma12152346

**Published:** 2019-07-24

**Authors:** Yangliu Du, Fuqiang Li, Yecan Peng, Shaowu Jia, Lei Lan, Jinghong Zhou, Shuangfei Wang

**Affiliations:** 1 School of Light Industry and Food Engineering, Guangxi University, Nanning 530004, China; 2 Guangxi Key Laboratory of Clean Pulp & Papermaking and Pollution Control, Nanning 530004, China

**Keywords:** Fe_3_O_4_/MnO_2_ nanocomposites, magnetic catalyst, photocatalysis, advanced treatment, bio-treated effluent of papermaking

## Abstract

Owing to the implementation of increasingly stringent water conservation policies and regulations, the pulp and paper mill industry must make increased efforts to meet the limits for pollutant emissions. The primary pretreatment and secondary biochemical treatment methods used currently generally fail to meet the country-specific environmental regulations, and the wastewater must be processed further even after being subjected to secondary biochemical treatments. In this work, we synthesized Fe_3_O_4_/MnO_2_ nanocomposites (FMNs) with a flower-like structure for use in the heterogeneous photocatalytic treatment of biotreated papermaking wastewater. FMNs1.25, which were formed using a KMnO_4_/Fe_3_O_4_ molar ratio of 1.25, could be separated readily using an external magnetic field and exhibited higher photocatalytic activity than those of the other samples as well as MnO_2_ and Fe_3_O_4_. The effects of various experimental parameters on the photocatalytic activity of FMNs1.25, including the initial pH of the wastewater and the catalyst dosage, were determined. The common chemical oxygen demand (COD_Cr_) reduction rate in the case of this sample reached 56.58% within 120 min at a pH of 3, the COD_Cr_ of effluent after treatment was 52.10 mg/L. Further, even under neutral conditions, the COD_Cr_ of the treated effluent was below the current limit for discharge in China. Moreover, the nanocomposites exhibited good recyclability, and their catalytic activity did not decrease significantly even after five usage cycles. This study should serve as a platform for the fabrication of effective photocatalysts for the advanced treatment of biotreated papermaking effluent and refractory organic wastewater.

## 1. Introduction

The effective treatment of wastewater plays an important role in maintaining the ecological balance of the natural environment [1,2,3,4,5,6,7]. Pulp and paper mills consume a lot of water and, at the same time, emit a considerable amount of wastewater [8,9]. Primary pretreatments and secondary biochemical treatments are the main steps in wastewater treatment [10,11]. Owing to the problems related to the scale, papermaking materials and bleaching methods used, wastewater recycling rate, wastewater treatment technology employed, and equipment renewal, some pollutants inevitably remain in the final discharged wastewater. In China, because of the increasingly strict environmental protection guidelines and the implementation of stricter regulations, it has become essential to subject wastewater to additional treatments. However, these advanced treatments for wastewater pose an urgent problem in case of the pulp and paper industry [12]. It is very important to remove the organic pollutants present in pulp and papermaking wastewater, as these compounds are refractory, toxic, mutative, and carcinogenic and can have long-term harmful effects on the ecological environment [13]. Photocatalytic oxidation techniques have many advantages. For instance, they involve mild and controllable reaction conditions, allow for the oxidative degradation of organic matter, and do not result in the secondary pollution of the environment. As a result, they are being studied widely [14,15,16].

MnO_2_, a typical transition metal oxide, is used extensively in batteries [17,18,19], supercapacitors [20,21,22], and photocatalysts [23,24,25,26,27] because of its special physical and chemical properties. Moreover, C_3_N_4_/MnO_2_ nanocomposites have been synthesized and used for the photocatalytic degradation of phenol and dye during water treatment [28]. Further, films of CuO/MnO_2_ nanorod arrays have been synthesized and used for the efficient catalytic oxidation of acid fuchsin dye [29]. Similarly, mesoporous MnO_2_ has been fabricated and utilized in the catalytic ozonation of 4-nitrophenol [30] while hierarchically structured MnO_2_@SiO_2_ nanofibrous membranes have been employed for the degradation of methylene blue [31]. These studies have shown that MnO_2_ has wide applicability as an efficient catalytic material. Nevertheless, MnO_2_ alone or nonmagnetic MnO_2_-containing heterogeneous catalysts cannot be separated and recycled readily after the treatment reaction. This increases the cost associated with their industrial use and also causes additional pollution [32]. Therefore, the development of novel catalysts with high separability and recyclability is essential. The magnetic separation technique provides a practical solution to this problem [33]. Loading MnO_2_ nanosheets onto the surfaces of Fe_3_O_4_ nanoparticles is an easy way of producing nanocomposite catalysts that can be removed and recycled readily using an external magnetic field. The durability of these catalysts is also high [34,35]. Zhang et al. [27] prepared a magnetic recyclable thin-layer MnO_2_ coated Fe_3_O_4_ nanocomposite by hydrothermal method combined with a mild ultrasonic means and used it in the photocatalytic decolorization of model pollutant MB (methylene blue) under ultraviolet irradiation. The results showed that the maximum photodegradation rate was 98.2% after UV-vis light irradiation for 3h. However, few studies have been done on the treatment of industrial wastewater by MnO_2_/Fe_3_O_4_ nanocomposite, especially in the advanced treatment of the biotreated effluent of papermaking wastewater, which has a complex composition, containing lignin, hemicellulose, residual alkali, sugars, inorganic salts, volatile acids, organic chlorides, etc.

In this study, hollow Fe_3_O_4_ nanospheres (HFNs) and flower-like Fe_3_O_4_/MnO_2_ nanocomposites (FMNs) based on these HFNs were synthesized through a simple process. First, the HFNs were fabricated through a hydrothermal method using different initial (NH_2_)_2_CO/C_6_H_5_Na_3_O_7_·2H_2_O molar ratios. Next, the FMNs were fabricated via the coprecipitation method using different KMnO_4_/Fe_3_O_4_ molar ratios. The fabricated HFNs had a raspberry-like surface, which allowed for a high catalyst surface area as well as the ready growth of the MnO_2_ nanosheets. The synthesized FMNs were subsequently used for the treatment of the biotreated effluent of papermaking wastewater. The effects of the molar ratios of the reactants on the morphologies of the HFNs and FMNs were evaluated. In addition, the effects of several other factors on the efficiency of the heterogeneous photocatalytic reaction were also studied in detail. Finally, the decrease in the common chemical oxygen demand (COD_Cr_) after the heterogeneous photocatalysis process and the recyclabilities of the photocatalysts were also investigated.

## 2. Materials and Methods 

### 2.1. Materials Used

Ferric chloride hexahydrate (FeCl_3_·6H_2_O), potassium permanganate (KMnO_4_), and urea ((NH_2_)_2_CO) were purchased from Guangdong Guanghua Sci-Tech Co. Ltd (Guangzhou, China). Sodium citrate (C_6_H_5_Na_3_O_7_·2H_2_O), sulfuric acid (H_2_SO_4_, 95%–98%), fuming hydrochloric acid (HCl, 37%), and ethanol (CH_3_CH_2_OH, ≥99.7%) were purchased from Tianjin Zhiyuan Chemical Co. Ltd. (Tianjin, China). Polyacrylic acid (30% solid, average M_w_: 3000) was purchased from Shanghai Macklin Biochemical Co. Ltd (Shanghai, China). All the reagents were analytical grade. Deionized water with a resistivity of 18.0 MΩ·cm^−1^ was obtained from a Milli-Q apparatus (Millipore, Bedford, MA, USA) and was used throughout the study. The wastewater used was the biotreated effluent from a factory of the Guangye Guitang Sugar Group Co., Ltd (Guigang, China). The pH of the wastewater was 6.58 and its COD_Cr_ was 120 mg·L^−1^.

### 2.2. Synthesis of Hollow Fe_3_O_4_ Nanospheres (HFNs)

The HFNs were synthesized by the conventional hydrothermal method [27], different reactant molar ratios were used in the synthesis experiment. First, 4 mmol of FeCl_3_·6H_2_O, different amounts (16, 18, and 20 mmol) of (NH_2_)_2_CO, and different amounts (8 and 12 mmol) of C_6_H_5_Na_3_O_7_·2H_2_O were dissolved in 60 mL of deionized water and stirred continuously for 30 min at room temperature. Then, 0.75 mL of polyacrylic acid was added to the resulting dispersion. After being constantly stirred for 30 min, the mixture was transferred to a 100 mL Teflon-lined autoclave and kept at 200 °C for 12 h. Next, the autoclave was cooled to room temperature, the products were washed with deionized water and ethanol three times, and finally dried at 60 °C in a vacuum oven. The HFN samples obtained were labeled as HFNs1, HFNs2, HFNs3, HFNs4, HFNs5, and HFNs6. The experimental parameters used to fabricate these samples are listed in Table 1.

### 2.3. Synthesis of Fe_3_O_4_/MnO_2_ Nanocomposites (FMNs)

The FMNs were synthesized through a modified coprecipitation process. First, KMnO_4_ (0.75, 1.5, 1.875, 2.25, or 3 mmol) was dissolved in 75 mL of deionized water. Then, 1 mL of fuming hydrochloric acid was added to the solution in a dropwise manner. After the mixture had been stirred for 30 min, 1.5 mmol of the as-prepared HFNs was dispersed evenly in the solution. Next, the mixture was stirred for 30 min at room temperature and heated to 90 °C and kept at that temperature for 90 °C for 3 h. The obtained brownish-black product was sequentially washed with deionized water and ethanol. Finally, the product was dried at 60 °C for 24 h in a vacuum oven. The synthesized FMN samples were labeled as FMNs0.5, FMNs1, FMNs1.25, FMNs1.5, and FMNs2, and the experimental parameters used to produce them are listed in Table 2.

### 2.4. Characterization

The X-ray diffraction (XRD) patterns of the FMNs were acquired using a Rigaku MiniFlex600 X-ray diffractometer (Rigaku, Tokyo, Japan) with a Cu-Kα radiation source. The measurements were performed at 40 kV × 15 mA and 2θ values of 5–80°. X-ray photoelectron spectroscopy (XPS) was used to determine the surface chemical compositions of the samples. The XPS analyses were performed using a Thermo ESCALAB 250XI spectrometer (Thermo Scientific, Waltham, MA, USA) with an Al-Kα radiation source. The C1s peak at 284.6 eV was used as the internal reference peak for calibration. Fourier transform infrared spectroscopy (FT-IR, Bruker Tensor II, Bruker, Karlsruhe, Germany) was also performed on the samples; KBr was used to form the tablets for the measurements. The surface morphologies of the samples were characterized using a ZEISS SIGMA HD field-emission scanning electron microscopy (FESEM) system (Zeiss, Oberkochen, Germany) and a FEI Tecnai G2 F20 transmission electron microscopy (TEM) system (FEI, Hillsboro, OR, USA). An LDJ 9600 vibrating sample magnetometer (LDJ Electronics, Dayton, OH, USA) was used to determine the magnetic properties of the as-prepared samples. The Brunauer–Emmett–Teller (BET) surface areas (m^2^ g^−1^) of the samples were measured at 77 K using a Micromeritics TriStar II 3flex automatic specific surface and porosity analyzer (Micromeritics Instruments Corporation, Norcross, GA, USA). The zeta potential of the suspension was recorded on a Malvern Panalytical NANO ZS90 (Malvern Ltd, Malvern, UK). The 3D-excitation-emission-matrix (3D-EEM) spectra of the treated wastewater samples were obtained using a Horiba FluoroMax-4 fluorescence spectrophotometer (Horiba, Kyoto, Japan). The excitation wavelengths were 220–440 nm, while the emission wavelengths were 300–600 nm. The COD_Cr_ values of the treated wastewater samples were measured using a COD/ammonia nitrogen double-parameter rapid tester (5B-3C(V8), Beijing Lianhua Technology Co. Ltd, Beijing, China).

### 2.5. Heterogeneous Photocatalytic Tests

**Figure 1:**
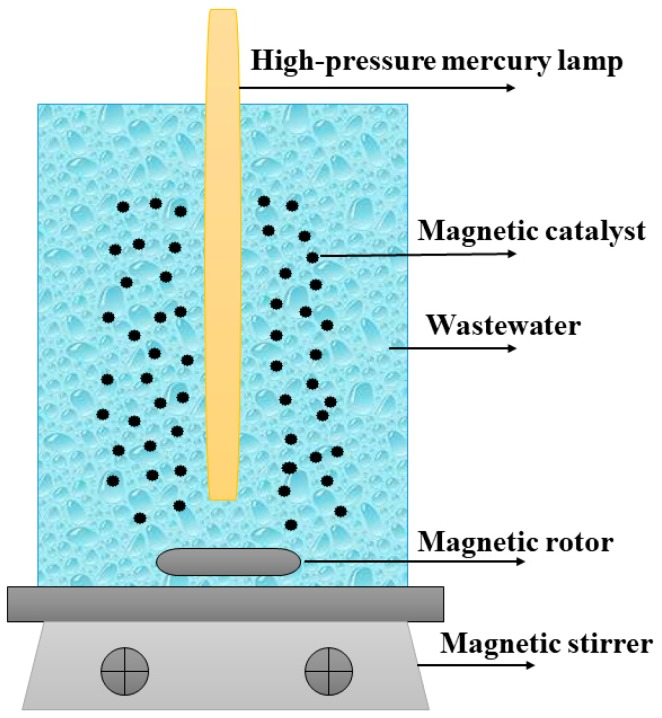
Schematic of reactor used for heterogeneous photocatalytic reaction.

The heterogeneous photocatalytic treatment of the secondary biotreated effluent of pulp and paper mill wastewater was performed in a GHX-V photochemical reactor (Shanghai Jiapeng Technology Co., Ltd., Shanghai, China); a schematic of the device is shown in Figure 1. The light source used was a 200 W high-pressure mercury lamp (wavelengths of 350–450 nm), and the reaction temperature was adjusted to 25 ± 2 °C. During each run, 250 mL of the wastewater was added to the reactor, and the initial pH of the reaction was adjusted using a 0.1 M NaOH or H_2_SO_4_ solution. Next, a certain amount of the FMNs sample in question was added to the solution. The suspension was stirred continuously and exposed to light. The COD_Cr_ value was determined by taking water samples at regular intervals. All the experiments were performed for same duration (180 min) in order to elucidate the effects of the photocatalytic treatment. Each group of samples was tested thrice, and the average COD_Cr_ was calculated. The COD_Cr_ removal rate of wastewater (α) can be calculated from the initial COD_Cr_ (C_0_) and the COD_Cr_ during sampling (C_t_) as follows:

α= (C_0_ − C_t_)/C_0_ × 100% (1)

## 3. Results and Discussion

### 3.1. Characterization of Hollow Fe_3_O_4_ Nanospheres and Fe_3_O_4_/MnO_2_ Nanocomposites

**Figure 2:**
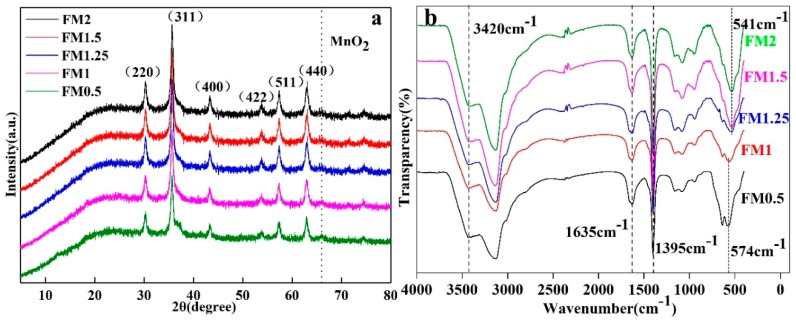
(**a**) XRD patterns and (**b**) FT-IR spectra of synthesized FMNs.

Figure 2a shows the XRD patterns of FMNs0.5, HFNs1, HFNs1.25, HFNs1.5, and HFNs2. The diffraction peaks at 2θ of 30.08°, 35.43°, 37. 02°, 43.08°, 53.42°, 56.95°, and 62.68° correspond to the (220), (311), (222), (400), (422), (511), and (440) reflection planes, respectively, and are consistent with the standard pattern of magnetite Fe_3_O_4_ (JCPDS Card No. 19-0629). Further, the diffraction peak appearing at 2θ of 65.98° is in keeping with the standard spectrum of γ-MnO_2_ (JCPDS Card No. 12–0714) and corresponds to MnO_2_ [36,37,38].

The FTIR spectra of the as-synthesized nanocomposites are shown in Figure 2b. In the cases of FMNs0.5 and FMNs1, the absorption peak at 574 cm^−1^ can be assigned to the Fe-O stretching vibrations that occur in iron oxide. Further, the characteristic peak at 3420 cm^−1^ is related to the stretching vibrations of the O-H bond and may be ascribable to the hydroxyl groups present on the sample surfaces. The peak at 1635 cm^−1^ corresponds to COO-stretching in citrates or peroxyacetic acid, while the peak at 1395 cm^−1^ can be attributed to the stretching of the C-O bond in citrates [39]. The peak at 458 cm^−1^ can be ascribed to the stretching of the Mn-O bond in MnO_2_ [40]. In addition, a peak related to the Fe-O stretching vibrations was also observed in the cases of FMNs1.25, FMNs1.5, and FMNs2 but at a lower wavenumber (541 cm^−1^), owing to an increase in the Mn-O bond strength [41].

**Figure 3:**
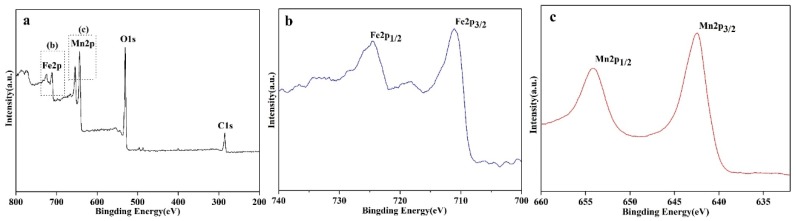
XPS survey spectrum of (**a**) FMNs1.25 and corresponding high-resolution (**b**) Fe2p, and (**c**) Mn2p spectra.

The chemical compositions and oxide states of the FMN samples were studied by XPS. The integral survey spectrum of FMNs1.25 is displayed in Figure 3a, which shows the binding energies of the Fe(2p), Mn(2p), O(1s), and C(1s) peaks of the nanocomposite. Figure 3b shows that two high-intensity bands with binding energies of 711.1 and 724.9 eV were present; these were the Fe2p_3/2_ and Fe2p_1/2_ peaks, respectively, and corresponded to the Fe^2+^ (FeO) and Fe^3+^ (Fe_2_O_3_) states, respectively, of Fe and were characteristic of the Fe_3_O_4_ structure [38,39,40]. Further, as can be seen from Figure 3c, the binding energies of the Mn2p_3/2_ and Mn2p_1/2_ peaks were 642.5 and 654.1 eV, respectively, indicating the presence of Mn^4+^ in the sample. These results were in keeping with previous reports [40,42]. Hence, these characterization data confirmed that the FMNs had been synthesized successfully.

**Figure 4:**
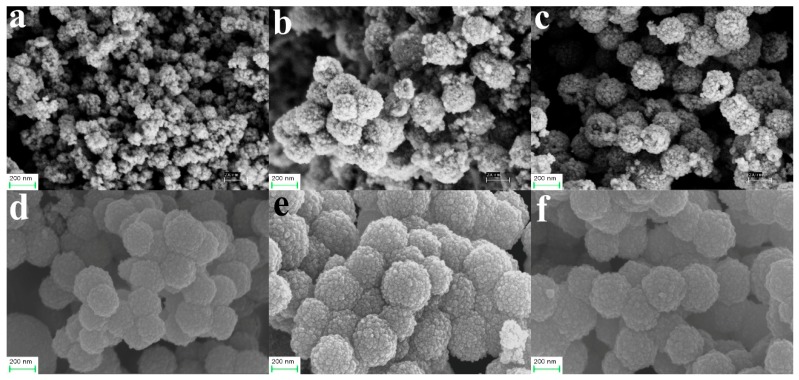
Field-emission scanning electron microscopy (FESEM) images of (**a**) HFNs1, (**b**) HFNs2, (**c**) HFNs3, (**d**) HFNs4, (**e**) HFNs5, and (**f**) HFNs6.

The dosages of (NH_2_)_2_CO and C_6_H_5_Na_3_O_7_·2H_2_O determined both the morphology and the size of the HFNs. Figure 4 shows FESEM images of the HFNs synthesized using the different initial (NH_2_)_2_CO/C_6_H_5_Na_3_O_7_·2H_2_O molar ratios. Figure 4a–c show the images of the HFNs produced using 8 mmol of C_6_H_5_Na_3_O_7_·2H_2_O. It can be seen from Figure 4a that the nanoparticles of sample HFNs1 had diameters of 20–30 nm. With an increase in the amount of (NH_2_)_2_CO added, as shown in Figure 4b,c, nanoparticles with rough surfaces and a diameter of approximately 200 nm were formed, as in the cases of HFNs2 and HFNs3. Moreover, a few incompletely formed spherical structures were also observed, indicating that the formed nanospheres were hollow. Figure 4d–f show FESEM images of the HFNs formed using 12 mmol of C_6_H_5_Na_3_O_7_·2H_2_O. It can be seen clearly that the HFNs had diameters of 200–300 nm and were well dispersed and fully formed. In summary, it can be concluded that the amount of C_6_H_5_Na_3_O_7_·2H_2_O used had a determining effect on the morphology and size of the HFNs formed. According to the molding conditions of the hollow nanospheres, HFNs6 sample was selected as the raw material for the subsequent experiments.

**Figure 5:**
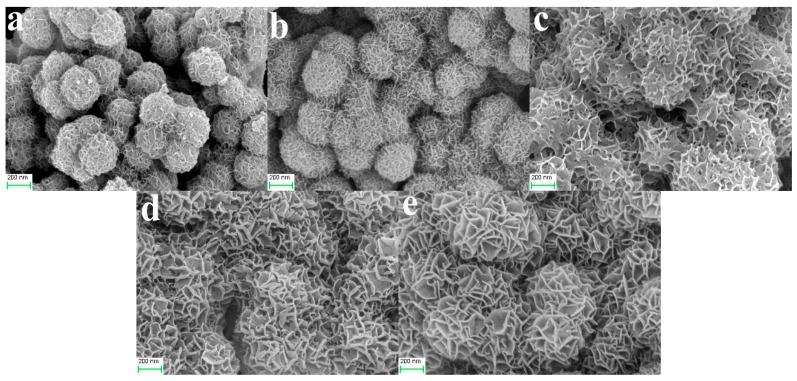
FESEM images of (**a**) FMNs0.5, (**b**) FMNs1, (**c**) FMNs1.25, (**d**) FMNs1.5, and (**e**) FMNs2.

**Figure 6:**
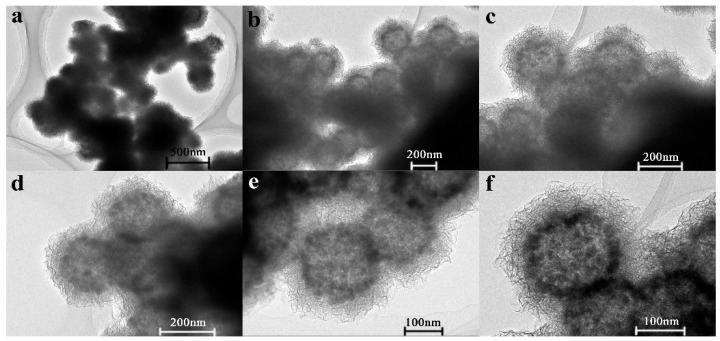
Transmission electron microscopy (TEM) images of FMNs1.25 at (**a**) 10,000×, (**b**) 15,000×, (**c**) 25,000×, (**d**) 30,000×, (**e**) 40,000× and (**f**) 50,000× magnification.

Next, the as-synthesized HFNs6 sample and different amounts of KMnO_4_ were used to prepare the various FMN samples by the coprecipitation method. The morphologies and microstructures of the fabricated FMNs can be seen from the FESEM and TEM images shown in Figure 5 and Figure 6, respectively. Figure 5 shows that, with an increase in the amount of KMnO_4_ added, the growth of the MnO_2_ nanosheets on the surfaces of the HFNs also increased. When the KMnO_4_/Fe_3_O_4_ molar ratio was increased to two, the MnO_2_ nanosheets turned into petal-like structures and completely covered the HFNs. This confirmed indirectly that the degree of bonding between the MnO_2_ nanosheets and the HFNs was high, resulting in well-structured nanocomposites.

The TEM images (Figure 6) also showed that FMNs1.25 consisted of nanospheres whose surfaces were completely coated with nanosheets. It can be seen from Figure 6b–e that the nanospheres had a black edge and a grayish-white central region. This confirmed that they were hollow.

**Figure 7:**
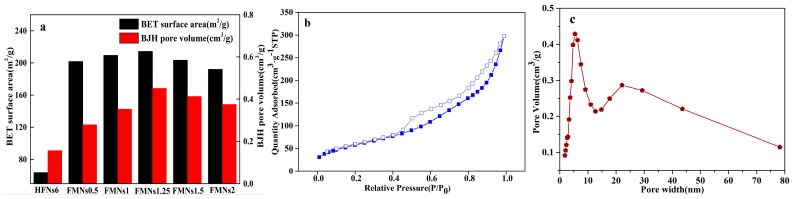
(**a**) Brunauer–Emmett–Teller (BET) surface areas and pore characteristics of HFNs6 and synthesized FMNs and (**b**) N_2_ adsorption–desorption isotherm and (**c**) pore size distribution of FMNs1.25.

The BET surface areas and pore characteristics of HFNs6 and the various FMNs are presented in Figure 7a. The BET surface area of pure Fe_3_O_4_ (HFNs6) was 63.69 m^2^/g, much lower than the value of MnO_2_ nanosheet. Further, with an increase in the loading rate of MnO_2_, the BET surface area of the nanocomposites (from FMNs0.5 to FMNs1.25) increased from 201.76 m^2^/g to 214.36 m^2^/g, much higher than the values of pure Fe_3_O_4_ due to the amount of MnO_2_ nanosheet. FMNs1.25 had the largest BET surface area at 214.36 m^2^/g. However, when the KMnO_4_/Fe_3_O_4_ molar ratio was increased to two, the BET surface area of FMNs2, was low at 192.00 m^2^/g, suggesting that excess KMnO_4_ solution could not increase the BET. The trend in the Barrett–Joyner–Halenda (BJH) pore volume of the nanocomposites was the same as that in the BET surface area, with FMNs1.25 exhibiting the highest BJH pore volume at 0.45 cm^3^/g.

The N_2_ adsorption–desorption isotherm of FMNs1.25 and its pore size distribution are shown in Figure 7b,c. There is significant hysteresis between the adsorption and desorption arms of the isotherm for P/P_0_ values between 0.4 and 1.0 (see Figure 7b); this was indicative of a typical mesoporous structure [43]. The pore size distribution of FMNs1.25 is given in Figure 7c. The distribution contains a sharp peak at 6 nm and a smaller peak at 22 nm. The average pore diameter was determined to be 8.32 nm. These data suggest that the fabricated catalysts had a high BET surface area and a mesoporous structure, which would result in the presence of a large number of active sites for the adsorbate. This, in turn, would promote the mass transfer of free radicals and hence dramatically improve the catalytic performance of the nanocomposites with respect to the organic pollutants present in wastewater [44,45].

**Figure 8:**
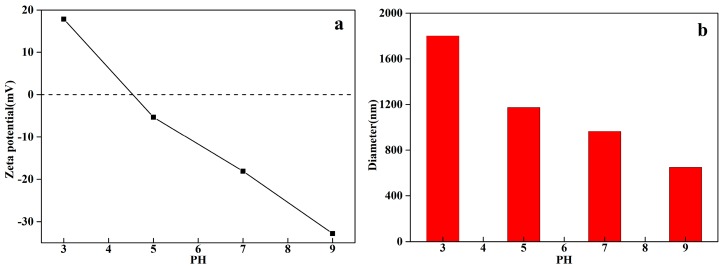
(**a**) Zeta potential and (**b**) diameter of FMNs1.25 as a function of PH.

The zeta potential and (b) diameter of FMNs1.25 were observed with the PH. As seen in Figure 8a, the Zeta potential of FMNs1.25 decreased with the rise of PH, the point of zero charge (PZC) of FMNs1.25 appeared between 3–5 of the initial PH of the reaction system, indicating the surface of FMNs1.25 was positively charged in solutions at PH below the PZC [46]. Therefore, the electrostatic attraction forces predominated at pH 3, leading to more organic pollutants adsorbing on the surface of catalysts and the COD_Cr_ removal rate reached higher. The average diameter varied as a function of pH between 3 and 9, and the results are shown in Figure 8b. The highest diameter of FMNs1.25 was 1801.1 nm at the PH of 3, when the PH of the solution was 9, the diameter reached it lowest point at 649.5 nm. These results show that the dispersion of catalyst in solution was getting better with the increases of PH.

### 3.2. Effects of Fe_3_O_4_/MnO_2_ Nanocomposites on Treatment of Wastewater Through Heterogeneous Photocatalysis

**Figure 9:**
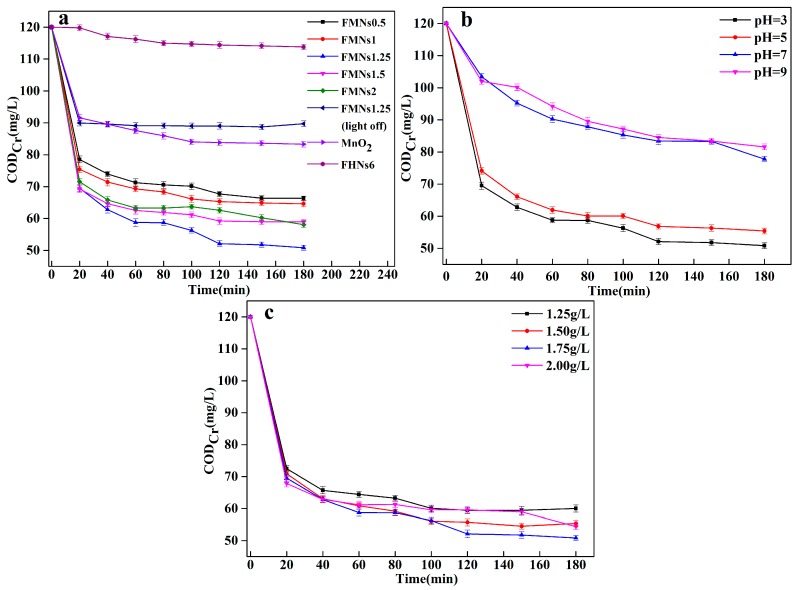
Effects of (**a**) FMNs sample, (**b**) initial PH, and (**c**) catalyst dosage on decrease in chemical oxygen demand (COD_Cr_) of wastewater.

A series of synthetic samples were applied to determine the COD_Cr_ removal efficiency, and the results are shown in Figure 9a. The areas of the curves before an elapsed time of 20 min represent the adsorption phase. It was obviously found that HFNs6 (pure Fe_3_O_4_) has lower COD_Cr_ removal efficiency compared with the Fe_3_O_4_/MnO_2_ nanocomposites under the same tested conditions. Control experiments performed using FMNs1.25 in the absence of UV-vis light showed that, on its own, the absorption of FMNs1.25 was 25.03%, the COD_Cr_ of effluent after treatment was 89.96 mg/L. There was no appreciable degradation of COD_Cr_ without light irradiation. The observed decreases in the COD_Cr_ in the case of the FMNs were principally attributable to the catalytic generation of radicals and the adsorption of organic pollutants by Fe_3_O_4_/MnO_2_ was limited. In contrast, after UV-vis light irradiation for 120 min, the maximal COD_Cr_ removal rates of FMNs0.5, FMNs1, FMNs1.25, FMNs1.5 and FMNs2 (reaction temperature of 25 °C, catalyst dosage: 1.75 g/L) were 43.58%, 45.57%, 56.58%, 50.66% and 47.83%, respectively. At the same time, the COD_Cr_ of effluent after treatment was 67.70 mg/L, 65.32 mg/L, 52.10 mg/L, 59.21 mg/L and 62.60 mg/L, respectively. The best COD_Cr_ removal rates (56.58%) were achieved by FMNs1.25, as indicated by the decreased COD_Cr_ concentrations from 120 mg/L to 52.10 mg/L, less than the prescribed level for discharge standard of water pollutants for pulp and paper industry in China. Previous studies have shown that under UV−vis light irradiation, the maximal degradation efficiency of methylene blue treated with a thin-layer MnO_2_ nanosheet-coated Fe_3_O_4_ nanocomposite could reach 98.2% [27] and the maximum adsorption capacities of As(III) and As(V) were 76.73 mg/g and 120.50 mg/g, respectively [47]. This means the Fe_3_O_4_/MnO_2_ nanocomposites have great potential, not only in the treatment of refractory industrial wastewater, but also in the treatment of heavy metal wastewater.

During the photocatalytic reaction, MnO_2_ plays a more important role than Fe_3_O_4_ [48]. The BET surface area and BJH pore volume of the FMNs increase with the growth of the MnO_2_ nanosheets. This, in turn, results in more active sites being present on the surfaces of the catalyst particles. Thus, the greater the amount of MnO_2_ in the nanocomposites, the higher their catalytic activity will be (as seen in the cases of FMNs0.5 to FMNs1.25). However, when the MnO_2_ loading amount was increased further (i.e., for FMNs1.25 to FMNs2), the BET surface area and BJH pore volume decreased, causing a decrease in the catalytic efficiency. Moreover, when MnO_2_ and Fe_3_O_4_ were used individually as catalysts for the heterogeneous photocatalytic reaction, the COD_Cr_ of the effluent remained higher than the current discharge standard in both cases.

Next, the effects of the pH and catalyst dosage on the decrease in the COD_Cr_ were investigated using FMNs1.25 as the photocatalyst. Figure 9b showed that the rate of decrease in the COD_Cr_ reduced as the pH was increased from 3.0 to 9.0. The highest decrease in the COD_Cr_ (57.63%) was achieved at a pH 3, the COD_Cr_ of treated wastewater was 50.84 mg/L. Further, the COD_Cr_ of effluent after treatment was 77.78 mg/L at a pH of 7 and 81.60 mg/L at a pH of 9. Hence, the efficiency of the catalytic reaction was significantly affected by the initial pH of the treated wastewater. As can be seen from Figure 8b, during the first 20 min, the adsorption capacity under acidic conditions (pH values of 3–5) was larger than that under alkaline conditions (pH values of 7–9). These results agreed with Zhang’s analysis [27], in that at acidic conditions, the surface of MnO_2_ nanosheet-coated Fe_3_O_4_ nanocomposite is positively charged, and then •OH production is accelerated. •OH production is greatly advantageous for the photocatalytic degradation of MB. The reactions involved in the above-described process are as follow [49]:

e^−^ + O_2_ →·O_2_^−^ (2)

e^−^ + H^+^ + O_2_^−^ → HO_2_^−^ (3)

HO_2_^−^ + H^+^ → H_2_O_2_ (4)

O_2_^−^ + H_2_O → HO_2_^−^ + OH (5)

The lower pH of reaction system, the higher concentration of H^+^ ions in the solution, causing the surfaces of the solid-phase catalyst particles to be positively charged. As a result, a greater number of the photogenerated electrons in the system migrate to the surfaces of the catalyst particles. This results in the combination of the electrons and the O_2_ that is adsorbed on the catalyst surfaces, leading to the generation of H_2_O_2_, this is consistent with Equations (2)–(4). Eventually, more ·OH is generated: Equation (5) represents the reaction process, resulting in an improvement in the efficiency of the catalytic reaction [49,50,51].

Thus, a pH of 3 was taken to be the optimal pH for the subsequent experiments to explore the effect of catalyst dosage on COD_Cr_ removal. Figure 9c shows the effects of the catalyst dosage on the rate of decrease in the wastewater COD_Cr_. When the catalyst dosage was increased from 1.25 g/L to 1.75 g/L, the decrease in the COD_Cr_ removal rate of wastewater increased from 49.97% to 57.63%. Further, when the catalyst dosage was increased to 2 g/L, the COD_Cr_ of the wastewater decreased by 54.67%. The particle size of the synthesized catalysts was small and the particles were well dispersed in the wastewater, resulting in an increase in the turbidity for the solution. Hence, some of the incident light was scattered and did not participate in the photocatalytic reaction. This adversely affected the catalytic efficiency [52,53].

From the above results, the as-synthesized catalyst shows high efficiency in the advanced treatment of wastewater from the pulp and paper mill. Moreover, it has been proved that a magnetically recyclable thin-layer MnO_2_ nanosheet-coated Fe_3_O_4_ nanocomposite performs well in the simulated dye wastewater [27]. In result, the Fe_3_O_4_/MnO_2_ nanocomposites in this study have great potential for the advanced treatment of biotreated papermaking effluent, dye wastewater and other refractory organic wastewater.

### 3.3. Kinetics of Heterogeneous Photocatalytic Reaction

**Figure 10:**
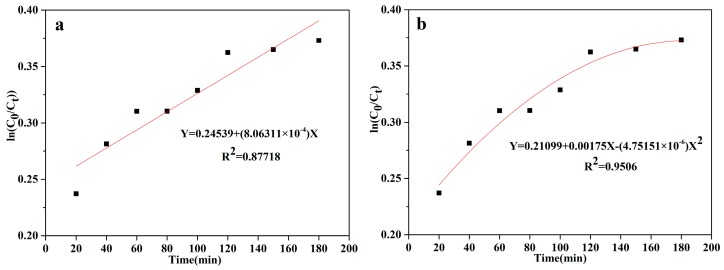
Fitted curves for (**a**) pseudo-first-order kinetics and (**b**) pseudo-second-order kinetics for decrease in COD_Cr_ during heterogeneous photocatalysis reaction (optimized conditions: Initial COD_Cr_ of 120 mg/L, FMNs1.25 concentration of 1.75 g/L, contact time of 180 min, solution pH of 3, and temperature of 25 °C).

The kinetics of the heterogeneous photocatalytic reaction were also studied. The software Origin 9.0 was used to fit the data corresponding to the relationship between the COD_Cr_ decrease rate and the reaction time. The reaction for the measurements was performed under optimized conditions, which were as follows: A reaction temperature of 25 °C, contact time of 180 min, with the following catalyst used: FMNs1.25, with an initial pH of 3, and a catalyst dosage of 1.75 g/L. Figure 10a,b show the pseudo-first-order-kinetics and pseudo-second-order-kinetics models obtained after multiple fittings. The fitting equation for the pseudo-first-order-kinetics model is Y = 0.24539 + (8.06311 × 10^−4^) X, with the pseudo-first-order-kinetics rate constant being 8.06311 × 10^−4^ and R^2^ = 0.87718. In addition, the fitting equation for the pseudo-second-order-kinetics model is Y = 0.21099 + 0.00175X − (4.75151 × 10^−6^) X^2^, with the pseudo-second-order-kinetics rate constant being −4.75151 × 10^−6^ and R^2^ = 0.9506. In the figures, the *y*-axis represents ln(C_0_/C_t_) while the *x*-axis represents the reaction time (min). These results indicated that the catalytic reaction was primarily driven by pseudo-second-order kinetics.

### 3.4. Recyclability of Fe_3_O_4_/MnO_2_ Nanocomposites

**Figure 11:**
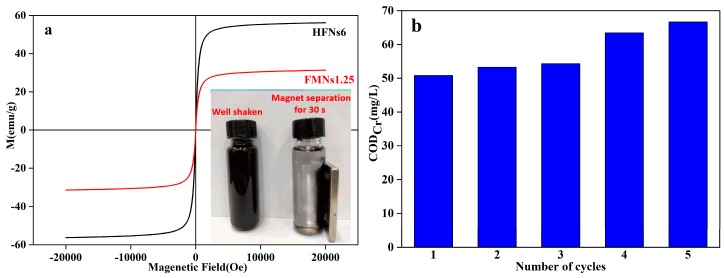
(**a**) Hysteresis loop of FMNs1.25 and (**b**) effect of number of reaction cycles on COD_Cr_ (optimized reaction conditions: Initial COD_Cr_ of 120 mg/L, FMNs1.25 concentration of 1.75 g/L, contact time of 180 min, solution pH of 3, and temperature of 25 °C).

With respect to heterogeneous photocatalytic reactions, the recyclability and reusability of the catalyst used are important parameters for evaluating its industrial applicability. The magnetic properties of the nanocomposites fabricated in this study had a significant effect on their reusability. As shown in Figure 11a, the saturation magnetization (MS) values of the HFNs6 and FMNs1.25 were 56.22 emu/g and 31.34 emu/g, respectively, when subjected to an external magnetic field. This allowed for solid-liquid separation within 30 s. The MS value of FMNs1.25 was lower than that of the HFNs6, owing to the presence of nonmagnetic MnO_2_ nanosheets, which limited its magnetization [27,47]. However, in spite of having a lower MS value, FMNs1.25 could be readily separated from an aqueous solution at a low magnetic field gradient (Figure 11a).

Reusability tests were performed, again under optimized conditions: Reaction temperature of 25 °C, the COD_Cr_ of the wastewater of 120 mg/L, contact time of 180 min, catalyst used: FMNs1.25, initial pH of 3, and catalyst dosage of 1.75 g/L. After each reaction, the photocatalyst used was separated from the wastewater sample being treated using an external magnetic field, and the collected catalyst was washed three times with water and ethanol respectively and dried at 60 °C for 24 h before the next experiment. The COD_Cr_ of the wastewater as measured after each test is shown in Figure 11b. It can be seen that, with an increase in the number of reaction cycles, the COD_Cr_ of wastewater after reaction decreased gradually. After the 5th cycle, COD_Cr_ reduced from the initial value of 120 mg/L to approximately 67 mg/L, which achieved 80% of the initial use effect. This confirmed that the catalyst exhibited good recyclability and reusability.

### 3.5. Degradation of Organic Pollutants

**Figure 12:**
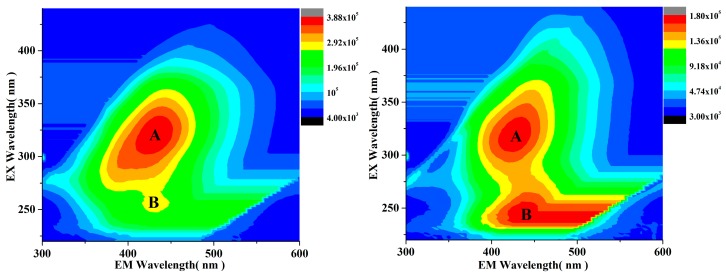
3D-excitation-emission-matrix (3D-EEM) spectrum of (**a**) untreated wastewater and (**b**) wastewater after catalytic treatment (optimized conditions: Initial COD_Cr_ concentration of 120 mg/L, FMNs1.25 concentration of 1.75 g/L, contact time of 180 min, solution pH of 3, and a temperature of 25 °C).

3D-EEM spectroscopy is a useful technique of analyzing the chemical properties of the organic compounds present in the wastewater, which can be evaluated based on the position, shift, and intensity of the fluorescence peaks present in the obtained spectrum [54]. Figure 12a shows the 3D-EEM spectrum of an untreated wastewater sample while Figure 12b shows the 3D-EEM spectrum of the wastewater sample after the heterogeneous photocatalytic reaction. In this case too, the reaction was performed under optimized conditions: Reaction temperature of 25 °C, contact time of 180 min, with the following catalyst used: FMNs1.25, with an initial pH of 3, and a catalyst dosage of 1.75 g/L. As can be seen from Figure 12a, two distinct fluorescence peaks (A and B) were present in the spectrum of the untreated wastewater sample. Peak A was centered at E_x_/E_m_ = 322 nm/435 nm and was the humic-related fluorescent peak in the visible region [55]. Furthermore, peak B was centered at E_x_/E_m_ = 246 nm/425 nm and displayed the fulvic-related fluorescent peak in the visible region [56]. After the photocatalytic reaction under optimized conditions, the center of peak A changed to E_x_/E_m_ = 318 nm/425 nm, while that of peak B moved to E_x_/E_m_ = 246 nm/440 nm (see Figure 12b). Moreover, the changes in the peak intensities were used to determine the differences in the contents of the various fluorescent components of the wastewater sample before and after the photocatalytic reaction. The intensity of peak A in the untreated sample was 3.88 × 10^5^ (a.u.) while that of peak B was 2.55 × 10^5^ (a.u.). After the heterogeneous photocatalytic reaction, the intensity of peak A decreased to 1.78 × 10^5^ (a.u.) while that of peak B decreased to 1.77 × 10^5^ (a.u.), indicating that the removal rate of the humic-like substance was 54.12% while that of the fulvic-like substance was 30.60%. In conclusion, while the heterogeneous photocatalytic reaction could remove more of the humic-like material than the fulvic-like material present in the wastewater sample, both components were degraded to a high degree.

## 4. Conclusions

To summarize, a series of magnetic, flower-like nanocomposites consisting of petal-like MnO_2_ nanosheets coated on hollow Fe_3_O_4_ nanospheres were synthesized by way of a simple hydrothermal method and a subsequent coprecipitation process. Sample FMNs1.25, which was fabricated using a KMnO_4_/Fe_3_O_4_ molar ratio of 1.25, exhibited higher photocatalytic activity than those of the other FMN nanocomposites as well as those of MnO_2_ and Fe_3_O_4_; this was the case even under neutral reaction conditions. The use of FMNs1.25 reduced the COD_Cr_ in the effluent to less than the prescribed level for discharge standard of water pollutants for pulp and paper industry in China, indicating that it is highly suitable for the photocatalytic treatment of wastewater. Further, because of their magnetic properties, the fabricated nanocomposite catalysts also showed good recyclability and reusability when used repeatedly in the treatment reaction. Thus, the Fe_3_O_4_/MnO_2_ nanocomposites fabricated have great potential for use as stable, efficient, environmentally friendly, and low-cost catalysts for the advanced treatment of refractory organic wastewater.

## Figures and Tables

**Table 1:** Experimental parameters used for synthesis of hollow Fe_3_O_4_ nanospheres (HFNs).

Sample	FeCl_3_·6H_2_O (mmol)	(NH_2_)_2_CO (mmol)	C_6_H_5_Na_3_O_7_·2H_2_O (mmol)	PAA (mL)	H_2_O (mL)
HFNs1	4	16	8	0.75	60
HFNs2	4	18	8	0.75	60
HFNs3	4	20	8	0.75	60
HFNs4	4	16	12	0.75	60
HFNs5	4	18	12	0.75	60
HFNs6	4	20	12	0.75	60

**Table 2:** Experimental parameters used for synthesis of Fe_3_O_4_/MnO_2_ nanocomposites (FMNs).

Sample	KMnO_4_ (mmol)	Fe_3_O_4_ (mmol)	HCl (mL)	H_2_O (mL)
FMNs0.5	0.75	1.5	1	75
FMNs1	1.5	1.5	1	75
FMNs1.25	1.875	1.5	1	75
FMNs1.5	2.25	1.5	1	75
FMNs2	3	1.5	1	75
